# Factors Associated with Home Health Aides’ Turnover Intention and Organizational Citizenship Behavior in Long-Term Care Services

**DOI:** 10.3390/healthcare10091743

**Published:** 2022-09-11

**Authors:** Wei Hsu, Fang-Chu Yang

**Affiliations:** Department of Health Care Management, National Taipei University of Nursing and Health Sciences, Beitou, Taipei 112303, Taiwan

**Keywords:** home health aides, turnover intention, organizational citizenship behavior, work engagement, job satisfaction, long-term care services

## Abstract

Background: The elderly and disabled population has rapidly increased in the world, and the demand for long-term care is also increasing. Home nursing care services are the main service demand. However, the high turnover rate of home health aides has led to a continuous shortage of staff, which affects the quality of care provided. Objective: This research established a model based on the theory of reasoned action to explore the relationships among home health aides’ work engagement, job satisfaction, turnover intentions, and organizational citizenship behavior for long-term care providers. Method: In this cross-sectional study, a structured questionnaire was sent to 455 participants, and 402 (response rate 88.4%) took part in the study. The goodness-of-fit test and path analysis of Structural Equation Modeling (SEM) was employed to test the proposed model. Results: Through the goodness-of-fit test of SEM, it was found that the data results have a good model fit. The results of path analysis displayed that home health aides’ work engagement and job satisfaction had a significantly negative impact on turnover intention and a significantly positive impact on organizational citizenship behavior; turnover intention had a significantly negative impact on organizational citizenship behavior. Conclusion: This research deduces the theory of reasoned action has sufficient explanatory power for the home health aides’ turnover intention and provides evidence that home health aides’ work engagement and job satisfaction reduce their turnover intention and promote organizational citizenship behavior.

## 1. Research Background

The aging population is a significant global issue. As people age, the incidence of disability rises, and the demand for long-term care services is also increasing. The home care service currently is one of the important types of long-term care and requires stable human resources. However, home care service is labor-intensive work, coupled with the public’s poor impressions of long-term care service personnel, such as low wages, minimal benefits, irregular hours of work, and unstable employment [1]. These are all restrictions on investing in long-term care services. Although many home health aides think long-term care services are very meaningful [2], there are still factors that contribute to higher turnover rates. The departure of home health aides exacerbates the shortage of care staff and further affects the effectiveness of care for service recipients [3]. In Taiwan, the Long-term Care plan 2.0 (LTC policy 2.0) was launched in November 2016 by the Executive Yuan, which extended both service items and population coverage and was meant to reinforce the current long-term care system; one of the challenges of LTC policy 2.0 is a lack of staff and caregiver support in the entire system [4].

The finite rational behavior is compatible with the assessment of information and the computational capacities that are actually possessed in the environments [5]. The theory of reasoned action, provided by Fishbein and Ajzen [6], was often used to explain the process of employee turnover. It stated that a person’s behavior could be influenced by an individual’s attitude toward the behavior through the intention to engage in a certain behavior [7]. Attitude refers to the perception and assessment of the performance of a particular behavior, including consideration of subsequent outcomes; the behavioral intention is the will to participate in a particular activity and is a prerequisite for actual execution [8]. Based on the rational behavior theory, the main concept of the model is based on workers’ attitudes, such as job satisfaction [9] and work engagement [10], as a predictor of employee turnover intention. In the current literature, there are few studies that use rational behavior theory as a theoretical model to predict home health aides’ turnover intention. In addition, explaining the relationship between employee turnover intention and organizational citizenship behavior has become an important research topic [11], but there is also a lack of literature on employee turnover intention and organizational citizenship behavior in the field of long-term care services. Therefore, this study aimed to analyze the impacts on home health aides’ work engagement, job satisfaction, turnover intention, and organizational citizenship behavior.

Work engagement is defined as a positive, fulfilling, work-related mental state characterized by “Vigor”, energy at work, mentally resilient, willingness to invest energy in work, and persevering even when encountering difficulties; “Dedication” is a sense of meaning, enthusiasm, inspiration, pride and challenge; and “Absorption” refers to full concentration at work, engaging with work, and feeling that time flies when working [12]. Job satisfaction is the pleasures derived from work, including the ability to make a positive impact on life through work [13]. Job satisfaction involves emotional, cognitive, and behavioral variables; emotional variables refer to work-related emotions such as exhaustion, nervousness, or pleasure; cognitive variables refer to beliefs about one’s own career, that is, the belief that one’s occupation is reasonably challenging and difficult; and behavioral variables include employees’ work-related behaviors, including tardiness, absence, or illness [14].

Turnover Intention is defined as a conscious and deliberate intention to leave the organization, and because it is highly relevant to actual separation, the intention to leave is often used as a representative of separation [15]. Intention to leave is a multi-stage process consisting of three parts, namely psychology, cognition, and behavior. Voluntary departures of employees are triggered by negative psychological reactions in the work environment that evolve into withdrawing cognitions and lead to actual departure behavior [16]. Work engagement is an important indicator of work attitudes [12], and higher employee engagement leads to higher happiness and physical and mental health and is associated with positive outcomes, such as higher job satisfaction [17] and lower intention to leave [18]. Job satisfaction has an impact on the desired work outcome and can be an important indicator of how employees feel about their work. It is influenced by salary, promotion opportunities, job security, or job requirements and has a negative impact on an employee’s intention to leave [19].

Organizational citizenship behavior is defined as the members’ behavior that is not formally recognized in the organization’s reward system but enhances the effectiveness of the organization as a whole. It is an act that supports the social and psychological environment in which task performance can unfold [20]. It is also an act that goes beyond an employee’s formal duties, including helping others, assuming other responsibilities, and promoting initiative [21]. As a discretionary act, organizational citizenship behavior usually consists of extra-role behavior, which is withdrawn when an employee intends to leave the organization because reducing organizational citizenship does not have any direct negative impact on the individual. The employee’s turnover intention hinders the development of their psychological attachment to the organization, which in turn hinders organizational citizenship behavior [22].

Work engagement is closely related to many important organizational outcomes, such as employees’ performance, organizational citizenship, and intention to leave [23]. Employees with high work commitment not only improve their work capacity and efficiency but also gain a competitive advantage and significantly improve organizational citizenship behavior [24]. Job satisfaction is the key to explaining employees’ attitudes and organizational citizenship behaviors [25] and is an important driver of organizational citizenship by enhancing employees’ willingness to work actively and stimulating employees to engage in beneficial out-of-role activities [26].

## 2. Method

### 2.1. Study Design and Conceptual Framework

This study was designed as a cross-sectional study by using a structured questionnaire. This research questionnaire was approved by the Research Ethics Committee of National Taiwan University (NTU-REC No. 202103ES009) and was classified into the Research Ethics Code. The survey was conducted anonymously, and the content of the answers was kept confidential to ensure the privacy of individuals and institutions. The data collected are for academic analysis purposes only. The subject objectives of this study are the home health aides of long-term care service institutions in Taiwan. Therefore, the home health aides of seven voluntary long-term care service institutions in Taiwan were issued the questionnaires. All the home health aides in the seven institutions were invited to participate in this study, regardless of their working experiences. With the consent of the institutions, the anonymous questionnaires were distributed at the monthly regular on-the-job training, and the distribution time was from May to August 2020.

**Figure 1:**
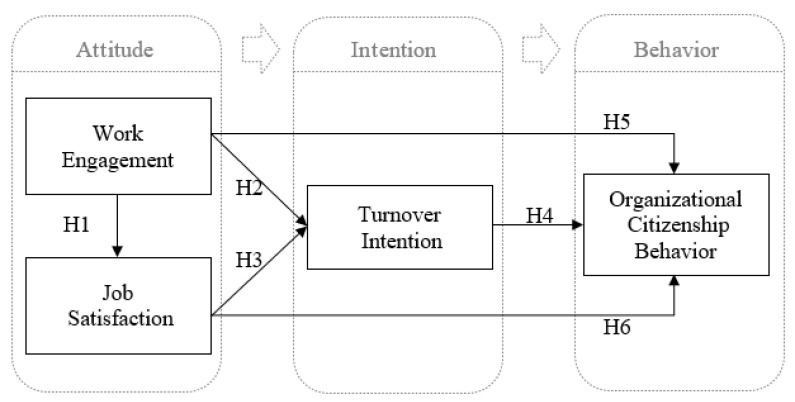
The Framework of Research Model.

This study constructed a research framework, as shown in Figure 1, based on the hypotheses of variable relationships from the literature review. Inferences from the above literature, this study proposed the following hypothesis:

**Hypothesis** **1** **(H1).** *Home health aides’ work engagement and job satisfaction are positively correlated*.

**Hypothesis** **2** **(H2).** *Home health aides’ work engagement and turnover intention are negatively correlated.*

**Hypothesis** **3** **(H3).** *Home health aides’ job satisfaction and turnover intention are negatively correlated*.

**Hypothesis** **4** **(H4).** *Home health aides’ turnover intention and organizational citizenship behavior are negatively correlated*.

**Hypothesis** **5** **(H5).** *Home health aides’ work engagement and organizational citizenship behavior are positively correlated*.

**Hypothesis** **6** **(H6).** *Home health aides’ job satisfaction and organizational citizenship behavior are positively correlated*.

### 2.2. Measurement

The content of the questionnaire refers to the relevant journal literature published in the past and modified according to the characteristics of Taiwan’s home health aides. There were five scholars and experts to review the meaning and relevance of the topic. The questionnaire content includes the respondent’s basic personal information and scales of work engagement, job satisfaction, turnover intention, and organizational citizenship behavior.

The work engagement scale, with a total of nine questions [12], was divided into three configurations, including “Vigor”, “Dedication”, and “Absorption”. “Vigor” included three items: “When I get up in the morning, I feel like going to work”, “At my work, I feel bursting with energy”, and “At my job I feel strong and vigorous”. “Dedication” had three items: “My job inspires me”, “I am enthusiastic about my job”, and “I am proud on the work that I do”. “Absorption” had three items: “I get carried away when I am working”, “I feel happy when I am working intensely”, and “I am immersed in my work”. This study measured home health aides’ work engagement by using a Likert five-point scale (strongly agreed = 5 to strongly disagree = 1). The overall Cronbach’s α coefficient of the work engagement scale is 0.95, and the measurement results have a very high degree of confidence.

Job Satisfaction Single Item (JSSI) has good measurement quality and makes it easier to assess satisfaction [27]. This study, drawing on Nielsen et al.’s [28] study, evaluated the overall job satisfaction of home health aides under the single question “How satisfied are you with current job?”, measured by the Likert’s five-point scale (strongly agreed = 5 to strongly disagree = 1).

This study referred to the study of Jang et al. [29] to measure turnover intention by an individual question. This study was adapted to the question, “If you had to decide whether to become a home health aide again, would you?”. In order to explore the turnover intention, this study scored the Likert’s five-point scale in reverse (strongly agreed = 1 to strongly disagree = 5).

Organizational citizenship behavior (OCB) was measured using the scales proposed by Ginsburg et al. [30] and Lee and Allen [31]. The scale measures two aspects of organizational citizenship behavior: the individual’s organizational citizenship behavior and the organization’s organizational civilizational citizenship behavior, for a total of twelve questions. All questions were measured by a Likert five-point scale (strongly agree = 5 to strongly disagree = 1). After removing the four questions where the factor is loading less than 0.60 (OCB2, OCB3, OCB4, and OCB11), there was a total of eight OCB questions. The overall Cronbach’s α coefficient of the organizational citizenship behavior was 0.90, representing a high degree of reliability of the scale.

### 2.3. Data Processing and Statistical Analysis

The research data were using descriptive analysis for sample characteristics, one-way ANOVA analysis and independent sample *t*-test for testing the difference of turnover intentions among personnel characteristics, and Structural Equation Modeling (SEM) for path analysis and goodness-of-fit of the model. In this study, the path analysis of potential variables is performed by standardized Maximum Likelihood Estimate (MLE) to test the coefficients of each path in the model.

## 3. Results

### 3.1. Research Results

In this study, the reliability and validity analysis of the measurement questionnaires of individual variables were processed. The results showed that Cronbach’s α for work engagement was 0.95 and the Cronbach’s α for organizational citizenship behavior was 0.90, both greater than 0.70, indicating a high reliability level. In terms of convergence validity, the standardized factor loading of each variable term ranged from 0.60 to 0.94. The composition reliability of the work engagement was 0.976, and the organizational citizenship behavior was 0.947, both greater than the requirements of 0.70. The average variation extraction (AVE) of work engagement was 0.932, and the organizational citizenship behavior was 0.646, both greater than the standard of 0.50, showing that measurements have convergence validity. The difference validity measures the degree of correlation between two different configurations, and if the correlation degree is low after analysis, it means the difference validity of the two masks. The square root of AVE of work engagement is 0.965, and the square root of AVE of organizational citizenship behavior is 0.804, both of which are greater than the correlation coefficients with other configurations. It can be seen that there is a difference in validity among the variables of the measurement.

A total of 455 questionnaires were issued, with 402 valid questionnaires, and the response rate was 88.4%. The respondents were mostly female, graduated from (vocational) high school, and were between 51 and 60 years old. This study processed the descriptive statistical analysis to analyze the data of 402 home health aides to understand the pattern of the sample in this study. It includes seven variables, including gender, age, education level, marital status, work experience, current job tenure, and full-time/part-time work. Table 1 displays the results of the descriptive statistical analysis. The home health aides of the research sample in this study were mostly women (353 (87.8%)); 51–60 years old (154 (38.3%)); had a high school education (192 (47.8%)); were married or had a cohabiting status (228 (56.7%)); had 5–10 years nursing assistant experience (138 (34.3%)); had 5–10 years current job tenure (133 (33.1%)); were in full-time work (382 (95%)). In terms of the seven variables of demographics, only turnover intentions of home health aides with different marital statuses had significant differences (*p* = 0.033).

Through the literature review, this study proposed a model to analyze the relationships among home health aides’ work engagement, job satisfaction, turnover intentions, and organizational citizenship behavior. The results of the offending estimate and normality test of the proposed model showed that the absolute values of skewness were less than 2 (between −1.225 and 1.149), and kurtosis of variables (between −0.444 and 2.519) was considered acceptable in order to prove normal univariate distribution. Mardia’s coefficient of work engagement, job satisfaction, turnover intention, and organizational citizenship behavior was 108.6, less than *p*(*p* + 2) of 195. It could be seen the population has a multivariate normal distribution. Standardized factor loadings were between 0.602 and 0.938, not over the threshold of 0.95, and the standard errors were between 0.006 and 0.023, so this model did not have an offending estimate.

As shown in Table 2, the standardized path coefficients for each potential variable can be seen, and work engagement significantly positively affects job satisfaction (*β* = 0.42, *p* = 0.001); H1 was supported. Work engagement significantly negatively affects turnover intention (*β* = −0.27, *p* = 0.001); H2 was supported. Job satisfaction significantly negatively affects turnover intention (*β* = −0.36, *p* = 0.001); H3 was supported. Turnover intention significantly negatively affects organizational citizenship behavior (*β* = −0.21, *p* = 0.001); H4 was supported. Work engagement significantly positively affects organizational citizenship behavior (*β* = 0.41, *p* = 0.001); H5 was supported. Job satisfaction significantly positively affects organizational citizenship behavior (*β* = 0.27, *p* = 0.001); H6 was supported.

The results of test of goodness-of-fit were shown that the five absolute fit indices were χ2/df = 2.099, GFI = 0.952, AGFI = 0.928, SRMR = 0.036 and RMSEA = 0.052. The five incremental fit indices were NFI = 0.957, NNFI = 0.970, CFI = 0.977, RFI = 0.945 and IFI = 0.977. Then, the three-model parsimony fit indices were PNFI = 0.748, PGFI = 0.638, and CN = 252. All of the indices of the model proposed in this study meet the standards, which means that the model fits the observations very well.

### 3.2. Discussion

The majority of the sample size were females, and the possible reason is that Taiwanese society traditionally believes that long-term care services are towards jobs suitable for females, so home health aides have a large disparity in the proportion of gender. The average age of the study sample was 54.5 years old, and the result might indicate that young people in Taiwan are currently less willing to engage in long-term home care services. At present, the age of home health aides in Taiwan is generally relatively old, and the foreseeable retirement wave may cause a shortage of long-term care staff. This once again highlights the importance of understanding home health aides’ turnover intentions.

The results revealed that home health aides in Taiwan have positive performance in work engagement, job satisfaction, and organizational citizenship behavior and have lower turnover intentions. The reason might be that middle-aged and elderly home health aides think that it is not easy to change jobs and have a high sense of identification with the organization. In this study, work engagement was significantly positively correlated with job satisfaction, which supported the hypothesis and was consistent with the previous study [17]. The results of this study show that there is a significant negative relationship between work engagement and turnover intention, which is consistent with the findings of many previous studies [18]. The higher the work engagement, the more home health aides, that is, the higher the dedication, energy, and focus, and the more loyal they are to the organization, which results in less turnover intention. The results also confirmed that dissatisfaction with work by home health aides would increase their turnover intentions [32].

It could be found there was a significant negative relationship between turnover intention and organizational citizenship behavior. Home health aides with higher turnover intentions were usually psychologically and emotionally detached from the organization, lacked motivation, were unwilling to focus their minds and energies on work, and were less likely to exhibit discretionary behavior [33]. Previous studies consistently showed a significant negative relationship between revolution intention and organizational citizenship behavior [34].

There was a significant positive relationship between work engagement and organizational citizenship behavior. When home health aides are more focused on their work, they are more likely to engage in selfless, conscientious, and ethical behavior. This finding is consistent with previous research, which showed high work engagement in the home health aides who recognize themselves as part of the organization, and significant effort is invested in achieving organizational goals and actively practicing organizational citizenship behavior [35,36].

The results of this study found that job satisfaction had a significant positive relationship with organizational citizenship behavior, consistent with previous studies [26]. Home health aides’ job satisfaction is a fundamental determinant of an individual’s behavior in an organization and is becoming increasingly important in motivating employees to act positively towards the company, including loyalty to the organization and organizational citizenship behavior [37].

Through the path analysis, the results of this study also found that home health aides’ work engagement has a greater impact on turnover intention and organizational citizenship behavior than job satisfaction. Work engagement could not only directly affect turnover intention and organizational citizenship behavior but also by improving home health aides’ job satisfaction, thereby reducing their turnover intention and increasing their organizational citizenship behavior. The impact of work engagement on turnover intention and organizational citizenship behavior is greater than job satisfaction, indicating the importance of increasing home health aides’ work engagement. Long-term care companies’ operational strategies, such as training home health aides in diverse skills, providing home health aides with competency development, expanding job roles, and giving learning opportunities, all contribute to home health aides maintaining a high level of work engagement. In addition, increasing work autonomy, task diversity, and the vision of support and development of colleagues and supervisors are also effective strategies for improving home health aides’ work engagement and job satisfaction. These strategies would be essential to ensure organizational stability, improve team cohesion, and care for quality.

## 4. Conclusions

The results of the study displayed that the proposed model has a high goodness-of-fit with the sample data. It is inferred that the theory of reasoned action could have sufficient explanatory power to interpret employee turnover intention and organizational citizenship behavior according to long-term care institutions. Home health aides’ work engagement could significantly improve their job satisfaction and reduce turnover intention, which in turn promoted organizational citizenship behavior.

The cross-sectional design of this study might limit the inference of causality between variables. Although the turnover intention is an important representative, the actual departure is still to predict. Employees who have a turnover intention do not always turn into actual departures, just as the behavior of turnover is relatively frequent, nor does it always express a turnover intention. It is therefore suggested that in the future, a longitudinal study could be conducted to analyze how various factors influence home health aides’ decisions over time, understanding the relationship between turnover intention and actual departure in the context of long-term home care services. Turnover intention is often influenced by a variety of variables, and this study explored home health aides’ turnover intentions only with work engagement and job satisfaction, suggesting that future studies can lead to an analysis of more other variables and assessing the presence of moderation or mediation effects between variables. A qualitative study of the motivations and experiences of these home health aides would be of great interest to continue with this topic for future study. Furthermore, the participant’s responses to this study, particularly some questions about the status of work and their current relationship with the institution, may also be influenced by societal expectations. The data in this study were not extended to other countries, which may affect the extrapolation of the results.

Academically, this study compensated for the lack of literature on home health aides’ turnover intention in the rational behavior theory, as well as the lack of relationships between work engagement, job satisfaction, revolution intention, and organizational citizenship behavior in the field of long-term care facilities. In practice, for residential long-term care service managers, understanding the factors influencing home health aides’ turnover intention will help develop strategies at the work and employee levels to support a more stable and high-quality care workforce. Managers need to proactively measure the available workforce, optimize the training of home health aides, attract competent workers to participate in training programs, and also train supervisors to improve support for home health aides and promote an organizational culture of mutual respect and appreciation, which will reduce turnover.

## Figures and Tables

**Table 1:** Analysis of the difference between personal characteristics and turnover intention (*n* = 402).

		Frequency (%)		Mean (SD)	*p*-Value
Gender ^a^	Female	353	(87.8)		0.124
	Male	48	(11.9)		
Age	≤30	8	(2.0)	54.5(9.7)	0.623
	31–40	24	(6.0)		
	41–50	95	(23.6)		
	51–60	154	(38.3)		
	>60	121	(30.1)		
Education ^a^	Elementary school and below	31	(7.7)		0.383
	National (Junior) Middle School	81	(20.1)		
	High school (vocational)	192	(47.8)		
	College	56	(13.9)		
	University and above	37	(9.2)		
Marital status ^a^	Married or cohabiting	228	(56.7)		0.033*
	Divorce or separation	76	(18.9)		
	Widowed	38	(9.5)		
	unmarried	54	(13.4)		
Work experience	Less than one year	50	(12.4)	7.8(6.1)	0.388
	1–5 years	104	(25.9)		
	5–10 years	138	(34.3)		
	More than 10 years	110	(27.4)		
Job tenure ^a^	Less than one year	66	(16.4)	6.9(5.7)	0.248
	1–5 years	113	(28.1)		
	5–10 years	133	(33.1)		
	More than 10 years	89	(22.1)		
Job status ^a^	Full time	382	(95.0)		0.732
	Part time	10	(2.5)		

^a^ Missing: Gender (*n* = 1), Education (*n* = 5), Marital status (*n* = 6), Job tenure (*n* = 1), Job status (*n* = 10).

**Table 2:** Path Analysis.

Hypothesis	Path	Hypothesis Relation	Estimate	*p*-Value	Test Result
H1	WE → JS	Positive	0.42	0.001	supported
H2	WE → TI	Negative	−0.27	0.001	supported
H3	JS → TI	Negative	−0.36	0.001	supported
H4	TI → OCB	Negative	−0.21	0.001	supported
H5	WE → OCB	Positive	0.41	0.001	supported
H6	JS → OCB	Positive	0.27	0.001	supported

## Data Availability

Not applicable.
